# Identification of plasma and urinary inflammatory markers in severe knee osteoarthritis: Relations with synovial fluid markers

**DOI:** 10.1186/s43019-024-00223-8

**Published:** 2024-05-21

**Authors:** Ji-Sun Shin, Hyobeom Lee, Seong Hyeon Kim, Kyu-Cheol Noh, Sung Jae Kim, Hyong Nyun Kim, Jae‑Young Choi, Si Young Song

**Affiliations:** 1 Department of Orthopaedic Surgery, Dongtan Sacred Heart Hospital, Hallym University College of Medicine, 7, Keunjaebong-gil, Hwaseong-si, Gyeonggi-do 18450 Republic of Korea; 2 Department of Orthopaedic Surgery, Kangdong Sacred Heart Hospital, 150, Seongan-ro, Gangdong-gu, Seoul, 05355 Republic of Korea; 3 Department of Orthopedic Surgery, Kangnam Sacred Heart Hospital, Hallym University College of Medicine, 1, Singil-ro, Yeongdeungpo-gu, Seoul, 07441 Republic of Korea; 4 School of Advanced Materials Science & Engineering, Sungkyunkwan University, Suwon, 16419 Republic of Korea

**Keywords:** Cytokine, Osteoarthritis, Plasma, Synovial fluid, Urine

## Abstract

**Background:**

This study aimed to identify plasma and urinary cytokines as potential biomarkers for severe knee osteoarthritis (OA). It also investigated associations between these cytokines and cartilage markers, as well as their connections with synovial fluid (SF) markers.

**Methods:**

Samples of plasma, urine, and SF were obtained from patients (*n* = 40) undergoing total knee arthroplasty (TKA) or unicompartmental knee arthroplasty (UKA) due to severe knee OA. Control samples of plasma and urine were collected from non-OA individuals (*n* = 15). We used a Luminex immunoassay for the simultaneous measurement of 19 cytokines, MMP-1, and MMP-3 levels. COMP, CTX-II, and hyaluronan (HA) levels were quantified using enzyme-linked immunosorbent assay (ELISA) kits. Receiver operating characteristic (ROC) curves were utilized to analyze each biomarker’s performance. Correlations among these biomarkers were evaluated via Spearman’s correlation.

**Results:**

The levels of plasma (p)CCL11, pCXCL16, pIL-8, pIL-15, pHA, urinary (u)CCL2, uCCL11, uCCL19, uCXCL16, uIL-1β, uIL-6, uIL-8, uIL-12p70, uIL-15, uIL-33, uMMP-3, uHA, uCTX-II, and uCOMP were significantly elevated in individuals with severe knee OA. Notably, specific correlations were observed between the plasma/urine biomarkers and SF biomarkers: pCCL11 with sfHA (*r* = 0.56) and sfTNF-α (*r* = 0.58), pIL-15 with sfCCL19 (*r* = 0.43) and sfCCL20 (*r* = 0.44), and uCCL19 with sfCCL11 (*r* = 0.45) and sfIL-33 (*r* = 0.51). Positive correlations were also observed between uCCL11 and its corresponding sfCCL11(*r* = 0.49), as well as between sfCCL11 and other cytokines, namely sfCCL4, sfCCL19, sfCCL20, sfIL-33, and sfTNF-α (*r* = 0.46–0.63).

**Conclusion:**

This study provides an extensive profile of systemic inflammatory mediators in plasma of knee OA and identified four inflammatory markers (pCCL11, pIL-15, uCCL11, and uCCL19) reflecting joint inflammation.

**Supplementary Information:**

The online version contains supplementary material available at 10.1186/s43019-024-00223-8.

## Background

Osteoarthritis (OA) is characterized by a high prevalence but lacks disease-modifying treatments before joint replacement surgery is resorted to. It is a growing global concern, attributed to aging populations, increasing obesity rates, and even sports-related injuries among young individuals [1]. OA requires long-term management after onset, so additional research is needed to identify and verify biomarkers [2]. Biomarkers are valuable for diagnosis, phenotype identification, disease progression prediction, treatment response monitoring, and predicting drug responsiveness in OA patients. Furthermore, these findings may help in understanding the pathogenesis of OA and developing new drug targets [3].

Currently, OA is understood to be a disorder affecting movable joints characterized by micro- and macro-damage that triggers maladaptive repair responses, including pro-inflammatory pathways. It is no longer regarded simply as a “wear and tear” disease confined to the older population [4]. Inflammation within the synovium is a consistent feature across all OA stages and is closely associated with symptomatic manifestations such as joint pain, swelling, and stiffness [5–7]. Various inflammatory cytokines, such as tumor necrosis factor (TNF), interleukins (ILs), and chemokines, play pivotal roles in mediating leukocyte infiltration and exacerbating irreversible cartilage degeneration [8–10]. Although inflammatory biomarkers for OA are expected to offer a promising avenue for revealing its pathogenesis and developing therapeutics [11], only a limited number of these biomarkers have been clinically applied.

Joint synovial fluid (SF) contains substances related to OA pathophysiology, including inflammatory mediators, cartilage-degrading enzymes, and cartilage-derived products; thus, it occupies an important position in biomarker research for OA. However, limitations in developing and clinically applying SF-based biomarkers exist due to the invasiveness of sample collection, variations in the presence of SF, and the skilled techniques required for collection [12]. Recently, blood or urine, which can be collected by a less invasive route, have been applied in biomarker research as a surrogate matrix for SF.

The aim of this study was to identify plasma and urine cytokines as potential biomarkers for severe knee OA and to analyze the correlation of these biomarkers with SF markers and cartilage turnover markers to contribute to the development of less invasive biomarkers for joint inflammation and degradation assessment. We selected 19 cytokines on the basis of previous reports related to the pathophysiology of knee OA [13–15]. Our study involved simultaneous assessment and comparison of 19 cytokines and five cartilage markers across SF, plasma, and urine samples from severe knee OA patients and controls. Given this focus, it was necessary to exclude participants who had conditions that could independently alter cytokine levels, as this would have confounded the relationship that we were trying to investigate between OA and cytokine levels. Conditions, such as rheumatoid arthritis, chronic renal diseases, chronic liver diseases, chronic obstructive pulmonary disease (COPD), malignant tumors, diabetes mellitus, and inflammatory bowel disease (IBD), are closely associated with inflammation. This association can affect their onset, progression, and the emergence of complications, leading to variations in systemic inflammatory markers and influencing cytokine levels [16–22]. Therefore, these conditions were excluded from this study, ensuring that the observed changes in cytokines had a direct relationship with OA disease.

## Methods

### Study design and population

This prospective case‒control study received approval from the institutional review board (IRB; No. HDT 2020-06-026), and all participants provided written informed consent. Between August 2020 and July 2021, patients who underwent total knee arthroplasty (TKA) or unicompartmental knee arthroplasty (UKA) for end-stage OA were recruited from our institution. The inclusion criteria were as follows: (1) a diagnosis of primary knee OA based on the clinical and radiological criteria of the American College of Rheumatology (pain in the knee and at least three of the following: age > 50 years, stiffness < 30 min, crepitus and osteophytes) [23] and (2) a disease severity grade 3–4 according to the Kellgren–Lawrence (K-L) classification. Exclusion criteria included: (1) traumatic arthritis, (2) rheumatoid arthritis, (3) chronic renal diseases, (4) chronic liver diseases, (5) chronic obstructive pulmonary disease (COPD), (6) malignant tumor, (7) diabetes mellitus, (8) inflammatory bowel disease (IBD), and (9) previous knee surgery on the same side. Age-matched individuals receiving regular medical examinations at the same hospital were recruited as healthy controls based on exclusion criteria. None of the control subjects had evidence of OA assessed clinically. Written informed consent was obtained from all patients and control subjects prior to inclusion in this study. Initially, informed consent was obtained from 60 participants (45 with severe knee OA and 15 healthy controls). Five patients with insufficient SF were excluded from the study, resulting in the inclusion of 55 participants (40 with severe knee OA and 15 healthy controls). The demographic data of the study population are presented in Table 1. This study exclusively analyzed and compared SF, plasma, and urinary inflammatory mediator levels in both the control group and knee OA patients, focusing solely on knee OA and not considering other variables.

**Table 1:** Demographic data of the study population

	Knee OA patients (*n* = 40)	Controls (*n* = 15)	*P*-value
Age, *years* (mean ± SD)	69.6 ± 6.8	65.4 ± 13.3	0.260
Height (cm)	155.0 ± 6.6	162.0 ± 12.1	0.113
Weight (kg)	64.1 ± 8.2	65.9 ± 10.4	0.528
BMI	26.6 ± 2.8	25.4 ± 3.4	0.189
Gender, *n*			
Women, *n* (%)	28 (70.0)	9 (60.0)	0.493
Men, *n* (%)	12 (30.0)	6 (40.0)	
K-L grade			
0, *n* (%)	0	11 (73.3)	
1, *n* (%)	0	4 (26.7)	
2, *n* (%)	0	0	
3, *n* (%)	6 (15.0)	0	
4, *n* (%)	34 (85.0)	0	
Surgery, *n*			
TKA, *n* (%)	36 (90.0)	–	
UKA, *n* (%)	4 (10.0)	–	

TKA, total knee arthroplasty; UKA, unicompartmental knee arthroplasty

### Biological sample collection and preparation

SFs (*n* = 40) were collected via needle aspiration from the patient’s knee joint during TKA or UKA surgery. A 16-gauge needle was connected to a 10 cc syringe for collection of SF. A standard anteromedial arthroscopy portal approach was taken to aspirate the contents of the joint. To ensure methodological consistency and minimize variability, the collection of SF from patients was conducted exclusively by a single surgeon. SF samples were centrifuged at 4000 × *g* for 10 min at 4 °C to separate solid debris and cells. The SF supernatant was aliquoted into microfuge tubes and stored at −80 °C until analysis. SF supernatant was subjected to analysis after reaction with hyaluronidase [24]. After overnight fasting, venous blood samples were collected from all participants into ethylenediaminetetraacetic acid (EDTA)-containing vacutainer tubes. Urine samples were collected by self-voiding in sterile urine collection cups with a screw-on lid. All participants were instructed to provide a midstream urine sample. Blood and urine samples were kept at 4 °C within 1 h of collection until processing. After separation by centrifugation (4000 × *g* for 10 min), plasma and urine supernatant were divided into aliquots into microfuge tubes and stored at −80 °C until analysis. The subsequent steps (collection, transportation, centrifugation, aliquot, and storage) of SF, plasma, and urine samples were uniformly handled by a single research staff member. This approach across all sample types was meticulously maintained to safeguard against any potential impact on sample quality or the study’s outcomes.

### Luminex bead-based assay

SF, plasma, and urine analytes were quantified using a Human Magnetic Luminex® Assay (R&D Systems Inc., Minneapolis, MN, USA) according to the manufacturer’s instructions [samples (1:2 dilution) or standards (7-point dilution)]. The 21-plex assay allowed us to evaluate the following analytes: C-C motif chemokine ligand (CCL) 2/MCP-1, CCL3/MIP-1α, CCL4/MIP-1β, CCL11/Eotaxin, CCL19/MIP-3β, CCL20/MIP-3α, C-X3-C motif chemokine ligand (CX3CL) 1/Fractalkine, C-X-C motif chemokine ligand (CXCL) 1/GROα, CXCL5/ENA-78, CXCL10/IP-10, CXCL16, interleukin (IL)-1β, IL-2, IL-6, IL-8, IL-12p70, IL-15, IL-33, matrix metalloproteinase (MMP)-1, MMP-3, and tumor necrosis factor (TNF)-α. The specific fluorescence was analyzed with a MAGPIX® system (Luminex Corp., Austin, TX, USA). The xPONENT 4.2 software package (Luminex Corp., Austin, TX, USA) was used for calibration, performance verification, and calculation of analyte concentration by using five-parameter logistic regression (5-PL).

### Enzyme-linked immunosorbent assay (ELISA)

Cartilage oligomeric matrix protein (COMP), C-terminal cross-linked telopeptides of type II collagen (CTX-II), hyaluronan (HA), and creatinine levels were determined using commercially available ELISA kits according to the manufacturer’s instructions (COMP, HA, and creatinine with R&D Systems Inc., Minneapolis, MN, USA, and CTX-II with Cusabio, Houston, TX, USA). Samples were diluted using reagent diluent to bring analyte levels into the calibration range of the assay as follows: COMP (1:40,000 dilution for SF, 1:800 dilution for plasma, and no dilution for urine), CTX-II (1:2 dilution for SF and urine and no dilution for plasma), and HA (1:100 dilution for SF, 1:5 dilution for plasma, and 1:2 for urine). The absorbance was analyzed using an Epoch microplate ELISA reader (Bio Tek Instruments, Winooski, VT, USA). All urinary marker levels were normalized to the urinary creatinine concentration.

### Statistical analysis

Analytes with more than 50% of the samples showing out-of-range low values were excluded. Supplementary Table S1 provides details regarding the assay sensitivity and detection rates for these analytes. For the statistical analyses, GraphPad Prism 8 (GraphPad Software, Inc., Boston, MA, USA) and SPSS Statistics 27 (SPSS, Inc., Chicago, IL, USA) were used. The results are displayed as the mean ± standard deviation (SD) or mean with 95% confidence interval (CI). The normality of the distribution was analyzed with the D’Agostino and Pearson omnibus normality test. Differences in analytes between patients and healthy controls were determined using the Mann‒Whitney *U* test (nonnormal distribution). A *P*-value less than 0.05 was considered to indicate statistical significance. The biomarker results were subjected to receiver operating characteristic (ROC) curve analysis to determine the sensitivity, specificity, and area under the curve (AUC). Optimal cutoff values were determined using Youden’s J statistic to determine the cutoff for knee OA in the control group. Spearman’s correlation coefficient was used to evaluate correlations between parameters. *P* < 0.05 was considered to indicate statistical significance. The sample size was determined using a priori power analysis with G*Power 3.1.9.4 software, focusing on the capacity of uCTX-II levels to differentiate knee OA patients from healthy controls [25, 26]. This analysis was based on the differences in uCTX-II levels reported by Jung et al. [27], where controls exhibited levels of 190 ± 109 ng/mmol and knee OA patients 429 ± 257 ng/mmol, resulting in an effect size (d) of 1.21. We set the allocation ratio of patients to controls at 3, with a power of 95% and an alpha at 0.05, and using a two-tailed test, which indicated a required sample size of 13 controls and 37 patients. Anticipating potential dropouts, we considered a dropout rate of approximately 10% for controls due to general attrition and 20% for patients, specifically influenced by the availability of sufficient SF. Thus, 15 (controls) and 45 (patient) subjects were assumed to be sufficient for the statistical analyses in this study. Ultimately, 5 patients were excluded due to insufficient SF, allowing the study to proceed with 15 controls and 40 patients.

## Results

### Increased cytokine levels in plasma, urine, and SF samples from patients with severe knee OA

**Fig. 1:**
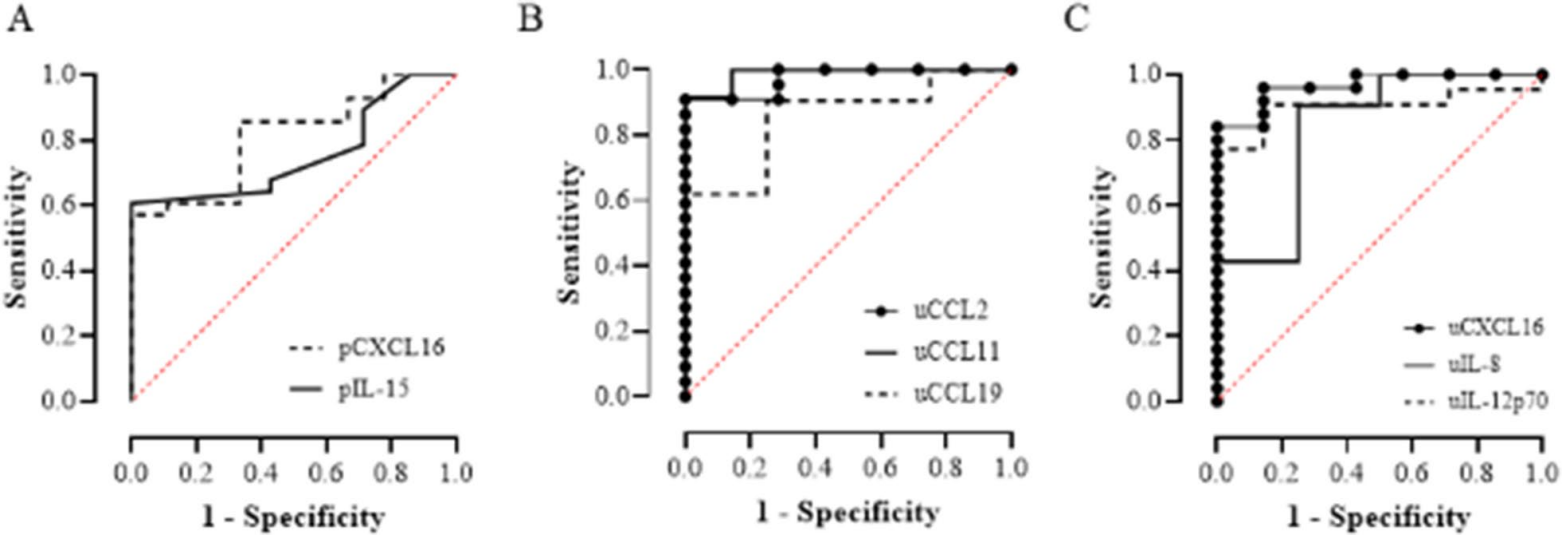
ROC curves for plasma and urine biomarkers in severe knee OA patients and controls. **A** pCXCL16 and pIL-15; **B** uCCL2, uCCL11 and uCCL19; and **C** uCXCL16, uIL-8, and uIL-12p70

This study enrolled 40 patients (40 knees) and 15 controls. Matched SF, plasma, and urine samples were collected from patients (*n* = 40; SF, plasma, and urine) and controls (*n* = 15; plasma and urine). The levels of 19 inflammatory cytokines in the plasma, urine, and SF were measured for severe knee OA patients and controls (Table 2). Significantly greater levels of pCXCL16 (*P* = 0.005) and pIL-15 (*P* = 0.038) were detected in severe knee OA patients. Table 3 listed the AUC values and the corresponding cutoff value, sensitivity, and specificity. The ROC curves generated for pCXCL16 and pIL-15 had AUC values of 0.81 and 0.76, respectively (Fig. 1).

**Table 2:** The levels of cytokines in OA patients and control groups

	Concentration (pg/ml), mean (95% CI)						
	Plasma			Urine			Synovial fluid
	Control	Knee OA	*P*-value	Control	Knee OA	*P*-value	Knee OA
CCL2	69.6 (42.2–97.1)	86.1 (72.4–99.9)	0.266	0.5 (0.2–0.8)	3.5 (1.8–5.2)	< 0.0001^***^	567.2 (419.0–715.4)
CCL3	NA	NA		NA	NA		51.4 (38.6–64.8)
CCL4	222.0 (173.7–270.4)	213.84 (192.3–235.4)	0.631	NA	NA		296.7 (271.7–321.8)
CCL11	NA	93.9 (76.4–111.4)		0.3 (0.15–0.38)	3.4 (−1.4–8.2)	< 0.0001^***^	93.6 (84.2–102.9)
CCL19	152.9 (17.4–288.3)	131.7 (36.2–227.3)	0.079	0.06 (−0.01–0.12)	0.3 (0.1–0.5)	0.002^**^	194.4 (91.2–297.6)
CCL20	NA	NA		NA	1.1 (−0.7–2.8)		97.3 (−46.8–241.5)
CX3CL1	1305.3 (1061.0–1550.0)	1266.6 (1125.0–1408.0)	0.512	8.4 (7.0–9.7)	11.9 (9.7–14.1)	0.104	6930.8 (5760.0–8101.7)
CXCL1	NA	NA		NA	NA		342.2 (−107.7–792.1)
CXCL5	511.1 (−40.7–1063.0)	388.9 (101.2–676.5)	0.569	NA	NA		171.9 (93.4–250.4)
CXCL10	33.7 (14.4–53.0)	42.3 (33.1–51.5)	0.224	0.06 (0.02–0.09)	0.1 (−0.01–0.3)	0.569	143.2 (88.1–198.3)
CXCL16	1052.7 (937.9–1167.0)	1314.0 (1214–1414)	0.005^**^	0.05 (0.02–0.08)	1.0 (0.4–2.0)	< 0.0001^***^	4467.4 (4131.9–4802.9)
IL-1β	NA	NA		NA	0.7 (−0.8–2.2)		NA
IL-2	NA	NA		NA	NA		3.4 (2.6–4.3)
IL-6	NA	NA		NA	0.5 (−0.06–1.0)		243.4 (−158.6–645.3)
IL-8	NA	3.1 (2.0–4.3)		0.05 (−0.05–0.2)	0.2 (0.09–0.4)	0.003^**^	103.1 (−22.0–228.2)
IL-12p70	NA	NA		0.7 (0.3–1.1)	9.7 (−4.0–23.3)	0.001^**^	NA
IL-15	2.8 (1.9–3.7)	4.4 (3.6–5.2)	0.038^*^	NA	0.5 (−0.4–1.4)		42.2 (38.4–46.0)
IL-33	NA	NA		NA	NA		4.27 (3.0–5.6)
TNF-α	1.2 (0.8–1.6)	1.7 (1.3–2.1)	0.108	NA	NA		2.1(0.9–3.3)

The concentrations of analytes were evaluated using a Luminex bead-based assay in plasma, urine, and synovial fluid from knee OA patients and controls

CI, confidence interval; NA, not applicable; ^*^P < 0.05, ^**^P < 0.001, and ^***^P < 0.001

**Table 3:** ROC analysis of plasma and urinary cytokines (controls versus knee OA)

	pCXCL16	pIL-15	uCCL2	uCCL11
AUC	0.81	0.76	0.97	0.99
95% CI	0.66–0.96	0.59–0.92	0.92–1.00	0.96–1.00
*P*-value	0.006^**^	0.03^*^	0.0002^***^	0.0001^***^
Cut-off (pg/ml)	1241	3.8	1.1	0.5
Sensitivity	0.57	0.61	0.91	0.92
Specificity	1.00	1.00	1.00	1.00

	uCCL19	uCXCL16	uIL-8	uIL-12p70
AUC	0.88	0.97	0.83	0.90
95% CI	0.68–1.00	0.91–1.00	0.58–1.00	0.79–1.00
*P*-value	0.026^*^	0.0002^***^	0.002^**^	0.002^**^
Cut-off (pg/ml)	0.1	0.2	0.06	1.6
Sensitivity	0.78	0.84	0.90	0.78
Specificity	1.00	1.00	0.75	1.00

CI, confidence interval; p, plasma; u: urine. ^*^P < 0.05, ^**^P < 0.001, and ^***^P < 0.001

Urinary levels of six proteins were notably greater in severe knee OA patients than in controls: uCCL2 (*P* < 0.0001), uCCL11 (*P* < 0.0001), uCCL19 (*P* = 0.002), uCXCL16 (*P* < 0.0001), uIL-8 (*P* = 0.003), and uIL-12 (*P* = 0.001). ROC analysis revealed AUC values of 0.97 for uCCL2, 0.99 for CCL11, 0.88 for CCL19, 0.97 for CXCL16, 0.83 for IL-8, and 0.90 for IL-12p70 (Fig. 1; Table 3). Our analysis revealed that, among plasma and urine markers, uCCL11 had the highest AUC for knee OA diagnosis, followed by uCXCL16.

Additionally, pCCL11, pIL-8, uCCL20, uIL-33, and uIL-1β were detectable in severe knee OA patients but undetectable in controls (Table 2). Elevated levels of CCL11, IL-8, IL-15, and CXCL16 were consistent in both plasma and urine samples from OA patients.

### Evaluation of cartilage markers in plasma, urine, and SF samples from severe knee OA patients

**Fig. 2:**
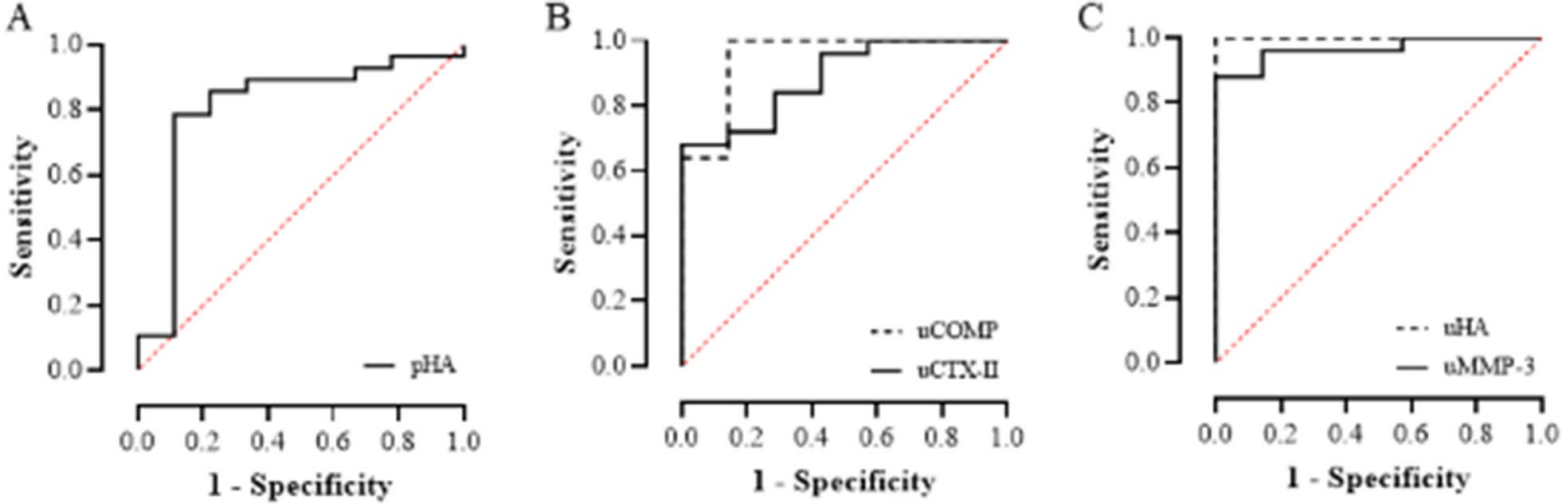
ROC curves for plasma and urine cartilage markers in severe knee OA patients and controls. **A** pHA, **B** uCOMP and uCTX-II, and **C** uHA and uMMP-3

Given the profound impact of inflammation on cartilage turnover, we investigated the potential link between inflammation and cartilage markers. We also evaluated the concentrations of five cartilage markers (COMP, CTX-II, HA, MMP-1, and MMP-3) in severe knee OA patients and controls (Table 4). The levels of cartilage markers in the OA SF samples were greater than those in the OA plasma or urine samples. The concentrations of pHA (*P* = 0.004), uCOMP (*P* < 0.0001), uCTX-II (*P* = 0.001), uHA (*P* < 0.0001), and uMMP-3 (*P* < 0.0001) were greater in severe knee OA patients than in controls. ROC analysis demonstrated AUC values of 0.81 for pHA, 0.95 for uCOMP, 0.89 for uCTX-II, 1.00 for uHA, and 0.97 for uMMP-3 (Fig. 2; Table 4).

**Table 4:** The levels of cartilage markers in the synovial fluid, plasma, and urine of OA patients or control groups

	Concentration, mean (95% CI)		*P*-value	ROC analysis (controls versus knee OA patients)					
	Control	Knee OA		AUC	95% CI	*P*-value	Cut-off	Sensitivity	Specificity
pCOMP, ng/ml	80.0 (64.1–96.0)	114.5 (87.9–141.1)	0.165						
pCTX-II, ng/ml	0.6 (0.3–0.9)	0.7 (0.5–0.9)	0.388						
pHA, ng/ml	23.3 (0.2–46.4)	55.4 (30.3–80.5)	0.004^**^	0.81	0.62–1.00	0.006^**^	20.3	0.77	0.89
pMMP-1, ng/ml	1.2 (−0.1–2.6)	0.9 (0.5–1.3)	0.903						
pMMP-3, ng/ml	13.5 (9.6–17.4)	15.0 (10.7–19.3)	0.520						
uCOMP, pg/ml	1.0 (−0.2–2.2)	5.8 (3.7–7.9)	< 0.0001^***^	0.95	0.85–1.00	0.0003^***^	1.1	1.00	0.87
uCTX-II, pg/ml	5.5 (2.2–8.8)	25.1 (12.4–37.7)	0.001^**^	0.89	0.76–1.00	0.002^**^	5.4	0.96	0.57
uHA, pg/ml	25.2 (14.4–36.0)	256.9 (192.9–320.9)	< 0.0001^***^	1.00	1.00–1.00	< 0.0001^***^	52.8	1.00	1.00
uMMP-1, pg/ml	NA	NA							
uMMP-3, pg/ml	0.5 (0.2–0.8)	3.5 (1.2–5.7)	< 0.0001^***^	0.97	0.91–1.00	0.0002^***^	0.9	0.88	1.00
sfCOMP, ng/ml	–	4553.6 (3557.8–5549.3)							
sfCTX-II, ng/ml	–	2.6 (2.3–3.0)							
sfHA, ng/ml	–	1314.5 (1238.0–1391.1)							
sfMMP-1, ng/ml	–	30.0 (15. 8–44.2)							
sfMMP-3, ng/ml	–	319.8 (285.5–354.0)							

The concentrations of analytes were evaluated using Luminex bead-based assay (MMP-1, MMP-3) or ELISA (COMP, HA, CTX-II) in plasma, urine, and SF from knee OA patient and controls. CI, confidence interval; NA, not applicable; p, plasma; u, urine; sf, synovial fluid. ^*^P < 0.05, ^**^P < 0.001, and ^***^P < 0.001

### Correlations between biomarkers in plasma samples and SF concentrations

We sought to identify plasma/urine biomarkers reflecting the osteoarthritic milieu by investigating correlations between these biomarkers and SF biomarkers. Spearman correlation analysis was performed on the plasma biomarkers (pCCL11, pCXCL16, pIL-8, pIL-15, and pHA) and urine biomarkers (uCCL2, uCCL11, uCCL19, uCCL20, uCXCL16, uIL-1β, uIL-6, uIL-8, uIL-12p70, uIL-15, uMMP-3, uHA, uCTX-II, and uCOMP) versus the overall biomarker profile of the SF samples. As shown in Table 5, pCCL11 exhibited a positive correlation with sfTNF-α (*r* = 0.58, *P* = 0.021) and sfHA (*r* = 0.56, *P* = 0.002). Conversely, pHA was negatively correlated with sfCCL11 (*r* = −0.47, *P* = 0.012). pIL-15 was positively correlated with sfCCL19 (*r* = 0.43, *P* = 0.024) and sfCCL20 (*r* = 0.44, *P* = 0.019).

**Table 5:** Correlation coefficient between markers

		*r*	95% CI	*P*
pCCL11	sfTNF-α	0.58	0.10–0.84	0.021
	sfHA	0.56	0.23–0.78	0.002
pIL-15	sfCCL19	0.43	0.06–0.70	0.024
	sfCCL20	0.44	0.08–0.70	0.019
pHA	sfCCL11	−0.47	−0.72–−0.10	0.012
uCCL11	sfCCL11	0.49	0.10–0.75	0.014
uCCL19	sfCCL11	0.45	0.03–0.73	0.032
	sfIL-33	0.51	0.12–0.77	0.012
uHA	sfCCL4	−0.54	−0.78–−0.17	0.005
	sfCCL19	−0.48	−0.74–−0.09	0.016
	sfCCL20	−0.46	−0.73–−0.07	0.019
	sfCXCL1	−0.50	−0.75–−0.11	0.012
	sfIL-15	−0.71	−0.86–−0.42	< 0.0001
	sfIL-33	−0.58	−0.80–−0.23	0.002
	sfMMP-1	−0.47	−0.74–−0.08	0.017
	sfMMP-3	−0.47	−0.73–−0.07	0.019
uCTX-II	sfCCL19	−0.43	−0.71–−0.03	0.032
	sfCXCL1	−0.53	−0.77–−0.16	0.006
	sfCXCL10	−0.41	−0.70–−0.004	0.042
	sfMMP-3	−0.48	−0.74–−0.09	0.016
sfCCL11	sfCCL4	0.48	0.12–0.72	0.009
	sfCCL19	0.52	0.17–0.75	0.004
	sfCCL20	0.46	0.11–0.71	0.011
	sfIL-33	0.51	0.16–0.74	0.005
	sfTNF-α	0.63	0.19–0.86	0.010

The *r*-Values represent Spearman correlation coefficients. Only correlations with statistical significance with a *P*-value < 0.05 are shown in the table. p, plasma; u, urine; sf, synovial fluid

### Correlations between biomarkers in urine samples and SF concentrations

As shown in Table 5, positive correlations between urine biomarkers and SF biomarkers were observed. uCCL19 was correlated with sfCCL11 (*r* = 0.45, *P* = 0.032) and sfIL-33 (*r* = 0.51, *P* = 0.012). Notably, among the systemic biomarkers, uCCL11 was the sole marker exhibiting a positive correlation with its corresponding sfCCL11 (*r* = 0.49, *P* = 0.014; Table 5) and with higher CCL11 concentrations in SF than in urine (Table 2). Intriguingly, sfCCL11 also exhibited positive correlations with five other SF cytokines: sfCCL4 (*r* = 0.48, *P* = 0.009), sfCCL19 (*r* = 0.52, *P* = 0.004), sfCCL20 (*r* = 0.46, *P* = 0.011), sfIL-33 (*r* = 0.51, *P* = 0.005), and sfTNF-α (*r* = 0.63, *P* = 0.010; Table 5).

Moreover, uHA exhibited a negative correlation with eight SF biomarkers (sfCCL4, sfCCL19, sfCCL20, sfCXCL1, sfIL-15, sfIL-33, sfMMP-1, and sfMMP-3), among which sfIL-15 (*r* = −0.71, *P* = 0.0001) exhibited the strongest negative correlation. uCTX-II was negatively correlated with four SF biomarkers (sfCCL19, sfCXCL1, sfCXCL10, and sfMMP-3), and sfCXCL1 (*r* = −0.53, *P* = 0.006) exhibited the most pronounced negative correlation. Furthermore, sfMMP-3 exhibited a negative correlation with both uHA (*r* = −0.47, *P* = 0.019) and uCTX-II (*r* = −0.48, *P* = 0.016; Table 5).

## Discussion

Biomarkers have the potential to revolutionize the quality of life of OA patients, improve individualized care, and help identify new therapeutic targets and mechanisms for more efficient drug trials [28]. This study aimed to identify inflammatory biomarkers, along with cartilage markers, in plasma and urine from knee OA patients. Furthermore, we examined the associations between systemic inflammatory markers (plasma and urine) and local joint inflammation (synovial fluid). Our main findings indicated elevated levels of several cytokines in plasma (pCCL11, pCXCL16, pIL-8, and pIL-15) and urine (uCCL2, uCCL11, uCCL19, uCCL20, uCXCL16, uIL-1β, uIL-6, uIL-8, uIL-12p70, and uIL-15) from severe knee OA patients. These cytokines emerged as effective identifiers of osteoarthritic patients, performing comparably to cartilage markers based on ROC analysis. And our results unveiled the correlations between systemic cytokines and cytokines/cartilage biomarker in SF among knee OA patients.

Among the cytokines tested, CCL11, IL-8, IL-15, and CXCL16 were consistently increased in both the plasma and urine. While direct comparisons of urine results are challenging due to limited reports on uCCL11, uIL-8, uIL-15, and uCXCL16, previous studies have reported elevated pCCL11 levels in knee OA patients compared with controls [29, 30]. Our data revealed bidirectional correlations between CCL11 and HA in plasma. pCCL11 exhibited a positive correlation with sfHA, whereas pHA displayed a negative correlation with sfCCL11. Notably, uCCL11 was the only cytokine showing a correlation with corresponding SF concentration, indicating its potential origin from the joint and its reflection of SF levels. sfCCL11 levels were positively related to K-L grade and the Western Ontario and McMaster Universities Arthritis (WOMAC) index, and knee OA patients with elevated sfCCL11 levels exhibited severe radiographic changes [30]. It was reported that CCL11 plays a crucial role in knee OA by being produced in cytokine-stimulated chondrocytes. Activation with CCL11 increased enzymes MMP-3 and MMP-13, linked to inflammation and cartilage damage. Furthermore, blocking CCL11 with an antibody significantly reduced MMP-3 expression triggered by IL-1β, pointing to a possible treatment approach for OA [30, 31]. Although our study did not confirm direct correlations between CCL11 and MMPs in urine or SF (Supplementary Table S2, S3), our findings revealed a positive correlation between sfCCL11 and several other SF cytokines (CCL4, CCL19, CCL20, IL-33, and TNF-α). Therefore, uCCL11 may be considered the most significant urinary cytokine and a biomarker reflecting intra-articular inflammation.

Previous reports have highlighted the correlation between sfIL-8 concentration and clinical severity [32], whereas serum IL-8 levels are reported to have no association with clinical severity [33]. It was reported that pIL-8 is positively correlated with pMMP-1 and pIL-15 in OA patients [34]. However, our study could not confirm these correlations. In our investigation, uIL-8 was positively correlated with uCX3CL1, uCXCL10, uCXCL16, uIL-6, and uMMP-3 (Supplementary Table S2). However, we found no SF markers that correlated with pIL-8 or uIL-8. IL-15 has been proposed as a potential biomarker for the early diagnosis of OA, with elevated levels observed in both SF and serum in early-stage disease compared with advanced disease [35, 36]. A correlation between sfIL-15 levels and sfMMP-1 and sfMMP-3 has been indicated [36]. Our study also revealed a positive correlation between sfIL-15 and sfMMP-1 (Supplementary Table S3). However, this correlation was not evident in the plasma samples.

In the field of biomarker research, cartilage markers have been the subject of comprehensive and in-depth studies. In line with the findings of previous studies [12, 37], our study reaffirmed the elevation of promising OA biomarkers, namely, pHA and uCTX-II, in severe knee OA. Additionally, we observed increased urinary levels of COMP, HA, MMP-1, and MMP-3 in severe knee OA patients compared with controls. There have been few reports on changes in the urinary levels of COMP, HA, MMP-1, and MMP-3 in OA patients. To assess the association of urinary cartilage markers with intra-articular inflammatory factors, we analyzed the correlation between plasma/urinary cartilage markers and SF cytokines. No significant positive correlations were detected between plasma or urinary cartilage marker levels and SF cytokine levels. uHA and uCTX-II levels were inversely related to alterations in important inflammatory and cartilage degradation markers in the SF. The strong negative correlation of uHA with sfIL-15, as well as the shared negative correlation of both uHA and uCTX-II with sfCCL19, sf CXCL11, and sfMMP-3, indicated their potential relevance in reflecting changes in the inflammatory milieu within the joint. These findings suggested that changes in uHA and uCTX-II levels may indicate successful modulation of inflammation and tissue degradation related to OA. Therefore, they have the potential to be useful as pharmacodynamic markers for assessing joint inflammation and monitoring responses to OA treatments. Further validation of these biomarkers in clinical studies is necessary to confirm their utility and establish their role in OA management.

Our findings contribute to the understanding of the inflammatory profiles in severe OA. The biomarkers identified in this study are associated with inflammatory processes that play a critical role in the pathogenesis of OA. These biomarkers could provide a deeper understanding of the biochemical and cellular processes involved in the progression of OA. They could inform the development of therapeutic strategies targeting inflammation in late-stage OA. These insights have the potential to be extended to early-stage OA in future research, offering a promising direction for developing diagnostic and monitoring strategies that are less invasive and broadly applicable across different stages of the disease.

This study has several limitations. First, biological samples were collected only from patients with K-L grade 3 or 4; therefore, we did not compare biomarker patterns between early-stage and end-stage OA. It was not possible to obtain blood and urine samples from patients with early-stage OA in our clinical setting. Second, it is still in the pilot stage with a relatively small number of patients. Third, SF was absent in the control group due to an insufficient amount of joint effusion present in healthy joints for aspiration; however, forcible collection can be considered unethical. Fourth, the phenotype of knee OA was not reflected. Knee OA is a disease with a large degree of heterogeneity, and it is known that clinical symptoms and related factors vary depending on the OA phenotype. Although this study considered inflammation as a major etiological factor of OA, there was a limitation in that the analysis subjects could not be limited to knee OA patients with an inflammatory phenotype. Finally, cytokines and cartilage turnover markers could not be analyzed for their association with radiological signs and clinical symptoms. Therefore, our results are provisional and need to be validated through further research to determine generalizability. Improving the study design and obtaining more data have the potential to enhance its practical value in clinical settings.

## Conclusion

We evaluated the correlations between multiple cytokines and cartilage markers in plasma, urine, and SF samples from severe knee OA patients. Fourteen cytokines and 5 cartilage markers were elevated in the plasma or urine of severe knee OA patients, compared with those in the control group. Several systemic biomarkers (pCCL11, pIL-15, uCCL11, uCCL19, uHA, and uCTX-II) were correlated with SF markers, suggesting their potential to reflect local synovial inflammation.

### Supplementary Information

Supplementary Material 1.

## Data Availability

The datasets used and/or analyzed during the current study are available from the corresponding author on reasonable request.

## Abbreviations

CCL: C-C motif chemokine ligand
CI: Confidence interval
COMP: Cartilage oligomeric matrix protein
CTX-II: C-terminal cross-linked telopeptides of type II collagen
CXCL: C-X-C motif chemokine ligand
CX3CL: C-X3-C motif chemokine ligand
ELISA: Enzyme-linked immunosorbent assay
HA: Hyaluronan
IL: Interleukin
MMP: Matrix metalloproteinase
ROC: Receiver operating characteristic
TNF: Tumor necrosis factor
TKA: Total knee arthroplasty
UKA: Unicompartmental knee arthroplasty
