# Supplementary material for: Identification of plasma and urinary inflammatory markers in severe knee osteoarthritis: Relations with synovial fluid markers
Source: Knee Surg Relat Res. 2024 May 21;36:19. doi: 10.1186/s43019-024-00223-8 (PMC11106897; doi:10.1186/s43019-024-00223-8)
Supplement: Supplementary file 1 — Supplementary Material 1. [file 43019_2024_223_MOESM1_ESM.docx]

**Supplementary Tables**

**Supplementary Table S1. List of investigated cytokines with minimal detectable concentration and percentage of detection rate**

| Analytes | Sensitivity | Detection rate (%) | | | | | |
| --- | --- | --- | --- | --- | --- | --- | --- |
|  |  | SF | Plasma | |  | Urine | |
|  |  |  | CON | OA |  | CON | OA |
| CTX-II | 0.08 ng/ml | 100 | 100 | 92.5 |  | 100 | 100 |
| COMP | 39.1 pg/ml | 100 | 100 | 100 |  | 100 | 100 |
| HA | 0.4 ng/ml | 100 | 100 | 100 |  | 100 | 100 |
| CCL2 | 9.9 pg/ml | 100 | 100 | 100 |  | 100 | 90 |
| CCL3 | 16.2 pg/ml | 80 | 13.3^*^ | 0^*^ |  | 0^*^ | 5^*^ |
| CCL4 | 5.8 pg/ml | 100 | 100 | 100 |  | 0^*^ | 10.0^*^ |
| CCL11 | 14.6 pg/ml | 100 | 93.3^*^ | 100 |  | 100 | 97.5 |
| CCL19 | 0.8 pg/ml | 100 | 100 | 100 |  | 73.3 | 92.5 |
| CCL20 | 3.4 pg/ml | 100 | 20^*^ | 27.5^*^ |  | 13.3^*^ | 52.5 |
| CX3CL1 | 64.8 pg/ml | 95 | 100 | 100 |  | 100 | 97.5 |
| CXCL1 | 5.3 pg/ml | 100 | 33.3^*^ | 50^*^ |  | 33.5^*^ | 37.5^*^ |
| CXCL5 | 8.2 pg/ml | 100 | 93.3 | 97.5 |  | 13.3^*^ | 37.5^*^ |
| CXCL10 | 1.2 pg/ml | 100 | 93.3 | 100 |  | 86.67 | 77.5 |
| CXCL16 | 0.4 pg/ml | 100 | 93.3 | 100 |  | 100 | 100 |
| IL-1β | 0.8 pg/ml | 42.5^*^ | 26.67^*^ | 15^*^ |  | 20^*^ | 67.5 |
| IL-2 | 1.8 pg/ml | 100 | 0^*^ | 12.5^*^ |  | 0^*^ | 5^*^ |
| IL-6 | 1.7 pg/ml | 97.5 | 13.3^*^ | 27.5^*^ |  | 20^*^ | 87.5 |
| IL-8 | 1.8 pg/ml | 100 | 13.3^*^ | 65 |  | 73.3 | 100 |
| IL-12 p70 | 20.2 pg/ml | 42.5^*^ | 0^*^ | 2.5^*^ |  | 100 | 87.5 |
| IL-15 | 1.0 pg/ml | 100 | 80 | 100 |  | 20 | 80.0 |
| IL-33 | 1.8 pg/ml | 100 | 13.3^*^ | 5^*^ |  | 20^*^ | 42.5^*^ |
| MMP-1 | 2.7 pg/ml | 100 | 100 | 100 |  | 13.3 | 40^*^ |
| MMP-3 | 5.3 pg/ml | 100 | 100 | 100 |  | 100 | 100 |
| TNF-α | 1.2 pg/ml | 92.5 | 60.0 | 87.5 |  | 0^*^ | 27.5^*^ |

^*^excluded from statistical analysis

**Supplementary Table S2. Correlation coefficient between urine markers**

|  | uCCL2 | uCCL11 | uCCL19 | uCCL20 | uCX3CL1 | uCXCL10 | uCXCL16 | uIL-1β | uIL-6 | uIL-8 | uIL-12p70 | uIL-15 | uMMP-3 | uHA | uCTX-II |
| --- | --- | --- | --- | --- | --- | --- | --- | --- | --- | --- | --- | --- | --- | --- | --- |
| uCCL11 | **0.69^c^** |  |  |  |  |  |  |  |  |  |  |  |  |  |  |
| uCCL19 | 0.40 | 0.42 |  |  |  |  |  |  |  |  |  |  |  |  |  |
| uCCL20 | 0.39 | **0.65 ^a^** | 0.47 |  |  |  |  |  |  |  |  |  |  |  |  |
| uCX3CL1 | 0.28 | 0.16 | -0.04 | 0.02 |  |  |  |  |  |  |  |  |  |  |  |
| uCXCL10 | 0.15 | 0.01 | -0.21 | 0.21 | **0.62 ^a^** |  |  |  |  |  |  |  |  |  |  |
| uCXCL16 | 0.14 | 0.12 | -0.06 | 0.16 | 0.30 | **0.63 ^a^** |  |  |  |  |  |  |  |  |  |
| uIL-1β | **0.55^a^** | 0.46 | 0.37 | 0.02 | 0.05 | 0.00 | 0.09 |  |  |  |  |  |  |  |  |
| uIL-6 | 0.09 | 0.31 | -0.04 | -0.15 | 0.26 | 0.55 | **0.67 ^b^** | 0.48 |  |  |  |  |  |  |  |
| uIL-8 | 0.41 | 0.20 | 0.17 | 0.35 | **0.54 ^a^** | **0.62 ^a^** | **0.61 ^b^** | 0.39 | **0.58 ^a^** |  |  |  |  |  |  |
| uIL-12p70 | **0.55 ^a^** | **0.63^b^** | **0.72^c^** | **0.72 ^a^** | 0.02 | 0.01 | -0.24 | 0.45 | 0.02 | 0.14 |  |  |  |  |  |
| uIL-15 | **0.79^c^** | **0.59^b^** | **0.57 ^a^** | 0.64 | 0.29 | 0.05 | 0.11 | 0.38 | 0.16 | 0.25 | **0.67^b^** |  |  |  |  |
| uMMP-3 | **0.44 ^a^** | 0.37 | 0.04 | -0.15 | 0.22 | 0.32 | 0.38 | 0.45 | 0.30 | **0.48^a^** | -0.03 | 0.04 |  |  |  |
| uHA | 0.07 | 0.05 | -0.33 | -0.42 | 0.04 | -0.11 | 0.01 | 0.29 | 0.10 | 0.06 | -0.05 | -0.21 | 0.14 |  |  |
| uCTX-II | 0.35 | 0.38 | 0.18 | -0.19 | 0.40 | -0.05 | 0.15 | 0.49 | 0.25 | 0.27 | 0.12 | 0.19 | 0.38 | **0.51 ^b^** |  |
| uCOMP | 0.31 | **0.50 ^a^** | 0.34 | 0.13 | 0.00 | -0.19 | -0.08 | 0.12 | -0.28 | 0.08 | 0.42 | 0.26 | 0.30 | 0.15 | 0.32 |

All r values represent Spearman correlation coefficients. The positive (p <.05) and negative (p <.05) associations are highlighted in bold. ^a^p <.05, ^b^p<.01, ^c^p<.001 (u: urine)

**Supplementary Table S3. Correlation coefficient between synovial fluid markers.**

|  | sfMMP-1 | sfMMP-3 | sfHA | sfCTX-II | sfCOMP |
| --- | --- | --- | --- | --- | --- |
| sfCCL2 | 0.04 | 0.18 | 0.06 | **0.74^c^** | 0.18 |
| sfCCL3 | -0.02 | -0.05 | 0.08 | **0.65^b^** | -0.04 |
| sfCCL4 | **0.54^b^** | **0.65^c^** | 0.10 | **0.41^a^** | 0.20 |
| sfCCL11 | 0.22 | 0.13 | -0.10 | 0.07 | 0.04 |
| sfCCL19 | **0.52^b^** | **0.64^c^** | -0.04 | 0.34 | -0.06 |
| sfCCL20 | 0.35 | **0.43^a^** | 0.18 | 0.35 | -0.25 |
| sfCX3CL1 | **0.66^c^** | **0.50^b^** | 0.13 | 0.21 | -0.38 |
| sfCXCL1 | **0.66^c^** | **0.61 ^c^** | 0.06 | **0.74^c^** | -0.02 |
| sfCXCL5 | **0.38** | 0.29 | -0.02 | **0.38^a^** | **-0.45^a^** |
| sfCXCL10 | **0.56^b^** | **0.58 ^b^** | 0.04 | **0.46^a^** | 0.26 |
| sfCXCL16 | 0.15 | 0.30 | -0.17 | 0.20 | 0.00 |
| sfIL-2 | **0.40^a^** | 0.38 | -0.01 | 0.37 | **0.50^a^** |
| sfIL-6 | **0.64^b^** | **0.65^b^** | 0.47 | **0.73^c^** | 0.04 |
| sfIL-8 | 0.11 | 0.20 | 0.04 | **0.66^c^** | -0.06 |
| sfIL-15 | **0.55^b^** | 0.42 | -0.06 | 0.34 | 0.02 |
| sfIL-33 | 0.31 | 0.23 | -0.01 | -0.15 | -0.08 |
| sfTNF-α | 0.47 | 0.42 | 0.12 | 0.23 | 0.23 |
| sfMMP-1 |  | **0.70 ^c^** | 0.02 | 0.37 | -0.23 |
| sfMMP-3 |  |  | 0.27 | **0.45^a^** | -0.07 |
| sfHA |  |  |  | 0.09 | 0.21 |
| sfCTX-II |  |  |  |  | 0.05 |

All r values represent Spearman correlation coefficients. The positive (p >.05) and negative

(p <.05) associations are highlighted in bold. ^a^p <.05, ^b^p<.01, ^c^p<.001
